# Perspectives on variability of *in vivo* toxicology studies: considerations for next-generation toxicology

**DOI:** 10.3389/ftox.2026.1778353

**Published:** 2026-03-02

**Authors:** Agnes L. Karmaus, Anna L. Kreutz, Oluwakemi Oyetade, Katie Paul Friedman, Martin Paparella, Emily N. Reinke, David Allen, Helena T. Hogberg, Nicole C. Kleinstreuer

**Affiliations:** 1 Inotiv, Research Triangle Park, Durham, NC, United States; 2 Independent Expert, Durham, NC, United States; 3 Institute for Medical Biochemistry, Medical University Innsbruck, Innsbruck, Austria; 4 National Toxicology Program Interagency Center for the Evaluation of Alternative Toxicological Methods, Division of Translational Toxicology, National Institute of Environmental Health Sciences, Durham, NC, United States

**Keywords:** generalizability, NAM evaluation, performance metrics, replicability, risk assessment, variability

## Abstract

**Introduction:**

Animal studies have historically informed toxicological testing and safety assessments. However, assessment of the variability in both quantitative and qualitative results has been limited. Biological variability, experimental differences, interpretation of categorical endpoints, and data availability and curation approaches all contribute to the quantified variability.

**Methods:**

A literature review was conducted to identify publications describing variability analyses for *in vivo* toxicology studies. Variability analyses were evaluated and summarized for a variety of toxicological endpoints: ocular irritation, dermal sensitization and irritation, acute oral and inhalation lethality, subchronic and chronic toxicity, carcinogenicity, neurotoxicity including DNT, endocrine, and genotoxicity.

**Results:**

This review summarizes published investigations of variability within mammalian toxicological studies that have been largely conducted in accordance with health effects test guidelines. The results of this review suggest that replicability of in vivo toxicological guideline studies varies widely by study type, endpoint complexity, and classification approach.

**Discussion:**

While any test system will have inherent variability, understanding its sources and impact on study interpretation will help ensure that appropriate confidence is applied when using the test method. Furthermore, such information aids in establishing relevant metrics to serve as baselines for informing performance characterization of new approach methodologies (NAMs). Future evaluation of NAMs should be contextualized using estimates of uncertainty and variance of the traditional study data to demonstrate “better” performance compared to traditional testing approaches. Robust understanding of guideline study performance is important for risk assessments, where it is important to find species-relevant NAMs that can perform at least as well as existing bioassays.

## 1 Introduction

Advancing the field of toxicology from animal models to new approach methodologies (NAMs) requires a robust understanding of animal study performance to ensure that the performance of NAMs is equivalent or better. “Equivalent or better” can be defined by many metrics, including but not limited to, more mechanistically insightful, more biologically relevant (e.g., human-based test systems for predicting human effects), and less variable as compared to the traditional animal-based test with respect to either replicate data themselves or the conclusions made for safety assessments. Herein we focus on characterizing variability of *in vivo* study outcomes by reviewing variability of replicate studies with quantitative and/or qualitative endpoints. Studies reviewed included different types of health effects guidelines, including ocular irritation, dermal sensitization and irritation, acute lethality, subchronic and chronic repeated dose toxicity, endocrine, carcinogenicity, neurotoxicity, developmental neurotoxicity (DNT), and genotoxicity tests. Characterizing the variability of data derived from guideline animal studies (or guideline-like, defined as having only minor variations from guideline study conduct) informs benchmarks for NAM performance with respect to NAM variability and replicability.

The concept of evaluating toxicological study variability is multifaceted, with different understandings and definitions dependent on where and how the concept of variability is applied. Evaluations of variability could consider differences in replicate study data, overall interpretation, and prediction of the toxicological outcome of interest. The National Academy of Sciences has defined the related term “reproducibility” in a programmatic manner, i.e., consistent results should be obtained when computational evaluations use the same input data, analysis steps, methods, code, and conditions of analysis (National Academies of Sciences E, 2019). Conversely, the National Academy of Science defines “replicability” as the concept of a repeated study outcome across multiple studies aimed at answering the same scientific question. The concept of data variability can also include “generalizability,” wherein a study outcome can be applied to other contexts or populations (e.g., species extrapolation for human health assessments and extrapolations from few model species to the diversity of species in the ecosystem) (Kukull and Ganguli, 2012). To clarify definitions used herein, we have summarized these terms in Table 1. The appropriate data and methods to evaluate variability and inform on whether a study is reproducible, replicable, and generalizable must be understood to gain insight into whether an existing method is reliable for a specific purpose and should be used to benchmark NAMs for the same or a similar endpoint. Our analysis primarily focused on replicability, as the available data included replicate studies derived from the same (or similar) guideline.

**TABLE 1:** Terminology for the different types of variability, as used in this manuscript.

Term	Explanation
Reproducibility	Variability of data from identical methods (e.g., a computational model)
Replicability	Variability of data from similar methods (e.g., test-protocol variability allowed within OECD TGs, including performance-based TGs or key-event-based TGs)
Generalizability	Variability of data from methods addressing the same endpoint for different species, populations or ecosystems

The standard approach for conducting toxicity testing for regulatory application is to utilize test guidelines. These can include Organisation for Economic Co-operation and Development (OECD) harmonized test guidelines, US Environmental Protection Agency (EPA, 2026) Health Effects Series 870 test guidelines, the International Council for Harmonisation of Technical Requirements for Pharmaceuticals for Human Use (ICH) guidelines, or International Organization for Standardization (ISO) testing standards. Such standardized test guidelines (TGs) provide acceptable study designs for international regulatory submissions. OECD harmonized TGs are intended to be robust, with emphasis on standardization of species, study length, exposure routes, and endpoints. However, OECD TGs are not standard operating procedures; they deliberately allow some protocol flexibility in terms of animal strains, exposure vehicles, experimental methods to analyze the endpoints, and statistics to assess the data. Study reports are still required to contain descriptions of how a study was conducted and to provide records of all aspects of the study. Further, regulatory agencies for all OECD member countries require the application of Good Laboratory Practice (GLP), which ensures detailed documentation of the quality and integrity of the study conduct. Adherence to GLP ensures proper storage of reagents and test articles, maintenance and accuracy of laboratory equipment, housing of animals, analytical approaches, test article characterization, and more (EPA, 2026). Studies conducted using a TG under GLP may provide sufficient documentation of a testing method to facilitate replicability analysis given shared study design parameters. One primary practical challenge is to gain access to well-documented study data and to then harmonize and curate them into databases that support retrospective analyses.

While adhering to TGs and GLP can support methodological consistency of *in vivo* guideline studies, inherent variability in terms of limited reproducibility, replicability, and generalizability is still frequently observed within and across studies. Toxicology is now at a critical time where toxicological data generated over decades are being curated for evaluation of their ability to inform appropriate benchmarks for NAMs that are often required to demonstrate performance that is “equivalent or better” when compared to traditional approaches. The availability of curated data has supported several analyses of variability across *in vivo* study types, but to date, these analyses have not been compiled and reviewed in one place. Ultimately, better understanding of *in vivo* variability is critical in leveraging NAMs for regulatory assessments as this understanding informs more realistic data-driven expectations for NAM assay performance. Therefore, we sought to aggregate available characterizations of variability from *in vivo* guideline study types. Rather than attempting to assess what the lowest theoretical variability from TGs optimally conducted and following the most recent scientific standards could be, this review is focused on curating the calculated replicability of existing TG data as they were generated and used for toxicological decision-making.

## 2 Materials and methods

### 2.1 Literature review

A literature review was conducted to identify publications describing variability analyses for *in vivo* toxicology studies. Initially, a literature search was conducted using PubMed (including MEDLINE) and Causaly databases. Medical Subject Heading (MeSH) terms and text words included “variability”, “reproducibility” and their variants combined with “*in vivo*”, “animal studies”, “experimental studies” and other relevant alternatives. The literature search focused on publications from 1990 to April 2023 (the date when the original search was conducted). This broad search, without restriction to toxicology, TG, or type of variability/reproducibility analysis yielded approximately 8,500 publications, confirming that evaluation of variability in the life sciences is a widely addressed topic.

Limiting to only publications containing variability analyses, systematic reviews, and meta-analyses of *in vivo* studies dramatically reduced the literature set. Subsequent manual screening, first by title and abstract, then subsequently by reviewing full text for a prioritized subset, was conducted to exclude non-relevant articles and adhere to the defined scope. This tiered manual screening resulted in the identification of approximately 100 relevant manuscripts; this corpus of literature was reviewed for data extraction and inclusion based on either quantitative analyses or relevant discussion addressing sources of variability in bioassays. This provided a set of about 60 manuscripts that included state-of-the-science reports containing opinions about scientific reproducibility of mammalian *in vivo* studies (not necessarily toxicology) and background on potential sources of variability that were used to inform our discussion.

From this body of literature, we selected toxicologically relevant study evaluations, specifically summaries of studies following human health-relevant TGs (from OECD and EPA Series 870 Health Effects). These guideline-like studies adhered to a set of criteria that were either aligned with existing regulatory guidelines or followed a standardized protocol used in regulatory assessment. We prioritized evaluations of data from mammalian studies, including analyses of variability from *in vivo* studies not specific to toxicology. The final number of publications yielding quantitative variability analyses that were useful for understanding replicability of relevant toxicological studies (e.g., TG or guideline-like) was 27. This subset was identified by excluding studies such as Ames assays that did not directly measure *in vivo* outcomes and endpoints such as metabolomics and vaccine evaluation that were considered too complex to evaluate for replicability.

### 2.2 Analyses from literature

Within our literature set, some reports evaluated variability in studies that were generally similar but did have minor differences (e.g., used different dosing vehicles). For those, we evaluated replicability among all studies, regardless of minor study differences. This was done for consistency, as not all analyses made such distinctions to account for study design. In studies that included multiple analyses, the most broadly representative summary metric was retrieved. For example, when respective analyses were conducted for males only, for females only, and for all animals, we considered only the evaluation that included all animals. Thus, our data compilation contains disparate data formats not intended to draw comparisons across study types; the intention of this variability review was to compile data and present reference values that can serve as a resource for better understanding *in vivo* toxicological studies.

Summaries of reported replicability were compiled for both categorical and quantitative endpoints. Variability analyses that considered categorical classification schemes were limited to those using the EPA and the United Nations Globally Harmonized System of Classification and Labeling of Chemicals (GHS) schemes. The replicability of European-specific classification, labeling, and packaging (CLP) criteria was not included in our review. Categorical replicability was generally reported in the literature set in the form of conditional probabilities. A conditional probability represents the probability of a chemical being assigned to a category given its prior categorization, with consideration to the number of studies with which a chemical was categorized. Calculations were performed as previously described (Karmaus et al., 2022; Luechtefeld et al., 2016).

Replicability of continuous quantitative endpoint values was reported as described by the primary literature source. We considered statistical metrics that conveyed some aspect of variance in replicate studies or the variance in replicate studies explained by study metadata (e.g., standard deviation [SD], coefficient of variation [CV], coefficient of determination [*R* ^2^], and root mean squared error [RMSE]; see Table 2).

**TABLE 2:** Summary of statistical metrics reviewed.

Statistical metric reported	General calculation	Relationship to replicability	Working definition
Percent replicable	The number of times a chemical resulted in that categorization from the total number of times the chemical was tested	100% indicates perfect replicability	Percentage of successful reproduction of a study categorization
Standard deviation (SD)	Calculated as the square root of the sum of squared deviations divided by the number of data points	Low SD indicates higher replicability	Measure of data dispersion around the mean in the same units as the data
Root mean squared error (RMSE)	Calculated as the square root of average squared distances between each actual and predicted value	Low RMSE indicates high model accuracy and low variance between predicted and actual values	Measure of model predictions from the true values (i.e., prediction error) in the same units as the data; when mean model prediction equals the data mean, the RMSE equals the SD of the model residuals
Coefficient of variation (CV)	SD divided by the mean, multiplied by 100	Low CV indicates low variability in the data with respect to the mean	Unitless value to indicate variability with respect to the mean; can be compared across datasets with different means but cannot be compared directly to SD or RMSE
Coefficient of determination (*R* ^2^)	*R* ^2^ = 1 - (SSE/TSS), where SSE is the sum of squared errors and TSS is the total sum of squares	Ranges from 0–1 with 1 being perfect replicability	Amount of variance in the data explained by a variable or model
Margin of uncertainty around the median	±2.5 x MAD (where MAD is the median absolute deviation)	A narrower margin indicates lower variability	A range of values in which the value of a parameter is expected to fall

## 3 Results

Published retrospective evaluations of *in vivo* toxicological guideline study replicability were retrieved and encompassed a variety of toxicological endpoints: ocular irritation, dermal sensitization and irritation, acute oral and inhalation lethality, subchronic and chronic toxicity, carcinogenicity, neurotoxicity including DNT, endocrine, and genotoxicity (Tables 3, 4). While the retrieved literature set included assessments of variability for study types that can be considered “complex” (i.e., carcinogenicity and DNT), relevant assessments were not found for other complex endpoints, such as offspring generation from prenatal developmental toxicity and multi-generation reproductive toxicity studies, delayed neurotoxicity, or toxicokinetics.

**TABLE 3:** Variability of measured categorical endpoints for *in vivo* toxicological studies.

Study type relevant TG	Observation	% replicable	Number of test articles	Number of studies	References
Ocular Irritation (Draize rabbit eye irritation test OECD TG 405^a^	GHS Cat	1: 73% 2A: 33% 2B: 16% NC: 94%	491	46 138 86 400	Luechtefeld et al. (2016) Table 3
	GHS Cat	1: 62.5% 2A/2B: 71.4% NC: 90%	42 16 7 20	89	Barroso et al. (2017) Table 4 and 5
	GHS Cat	1: 92.8%^b^ 2A/2B: 88.2% NC: 99.9%	NA	1860 studies, 582 animals 128 193 1,536	Adriaens et al. (2014) Table 10NCD
Dermal Sensitization (LLNA) OECD TG 429^a^	EC3 Cat	NS: 80% Weak: 68% Moderate: 63% Strong: 58% Extreme: 92%	38 8 8 12 4 6	333 61 38 128 57 49	Hoffmann et al. (2005) Table 1
	GHS Cat	1A: 69% 1B: 68% NC: 52%	87 36 65 35	400	Dumont et al. (2016) Table 3
Dermal Irritation/Corrosion OECD TG 404^a^	GHS Cat	1: 86% 2: 64% 3: 45% 4: 92%	425	1,065 endpoint study records 207 35 133 690	Rooney et al. (2021) Table 1D
Acute Lethality (oral LD50) OECD TG 420^a^	GHS Cat	54% (based on modeled LELs)	97	1,060	Hoffmann et al. (2010) Table 2
	EPA Cat	1: 53% 2: 49% 3: 62% 4: 66% 5: 75% I: 58% II: 67% III: 80% IV: 55%	2,241 53 183 556 1,663 1,490 236 910 2,341 458	7,574 104 342 1,166 395 2,857 446 1,694 4,648 788	Karmaus et al. (2022) Table 4
Acute Lethality (inhalation LC50) OECD TG 433^a^	EPA Office of Pesticide Programs categorization	I: 70% II: 68% III: 47% IV: 86%	339	75 137 100 556	Hull et al., *in prep*
Subchronic/Chronic Repeated Dose OECD TG 407^a^	% concordance of findings in Subchronic or Chronic	38.5%–90% (mean: 69%) depending on species, study type, and organ	169–538	306–2,170	Paul Friedman et al. (2023) Figure 2 and Supp Table 2
Carcinogenicity (chronic testing) OECD TG 451^a^	Pos/Neg	65% between rat sexes and 36% between species (rat and mouse)	313	379 (349 in rat, 339 mice)	Haseman and Lockhart (1993) Table 7 & 9
		86% between sexes 74% between species (rat/mouse)	​	379	Huff et al. (1991) Table 4
	GHS categorization	<50% for tumors in same GHS category	121	​	Gottmann et al. (2001) Table 7
Hershberger OECD TG 441^a^	Pos/Neg	72%	25	≥2 studies per chemical	Browne et al. (2015) Supp Table 6
Uterotrophic OECD TG 440^a^	Pos/Neg	74%	118	458 studies	Kleinstreuer et al. (2016), Table 2
Genotoxicity	Pos/ambiguous/Neg	78%–23%, depending on TG	13 to 78, depending on TG	3 to 23 replicates per substance, depending on TG	Raitano et al. (2026)

^a^ OECD, test guideline numbers represent the assay focus, however, several of the references include aggregated datasets including “guideline-like” studies with minor variations from the TG.

^b^ Values reflect modeled LELs, not direct experimental data.

Definitions–Carc, carcinogen; Cat, category; EC3, effective concentration required to induce a three-fold upregulation of lymph node cell proliferation; NA, not available; NC, not categorized; Non-Carc, non-carcinogen; Neg, negative; Pos, positive; TG, test guideline.

**TABLE 4:** Replicability of measured continuous endpoints for *in vivo* toxicological studies.

Study type/Relevant TG	Endpoint measure	Variability^a^	Number of test articles	Number of studies evaluated^b^	References
Ocular Irritation (Draize rabbit eye irritation test) OECD TG 405^c^	MAS	Interlaboratory CV: 42%–59%	9	24 labs	Weil and Scala (1971), Earl et al. (1997)
	MAS	Intralaboratory CV: 38%	​	4 labs, 13 tests	Earl et al. (1997), Cormier et al. (1996)
	MAS	Intralaboratory CV: 3%–65%	4	2 labs, 5 occasions	Earl et al. (1997), Blein et al. (1991)
Dermal Sensitization (LLNA) OECD TG 429^c^	LogEC3	SD: 0.147 logEC3 values	12	94 assays	Roberts et al. (2016)
Acute Lethality (Oral LD50)	LogLD50	SD: <0.42 log (mg/kg)^d^ Rat-Mouse Interspecies *R* ^e^: 0.80	57 40	504 studies 622 values	Hoffmann et al. (2010)
OECD TG 420^c^	LogLD50	Margin of uncertainty^e^: 0.095 ± 0.24 log (mg/kg)	1885	5,826 studies	Karmaus et al. (2022)
Subchronic/Chronic Oral Repeated Dose OECD TG 407^c^	LEL (study-level)	Full dataset LEL^f^: RMSE 0.589 log10-mg/kg/day	563	2,724	Ly Pham et al. (2020) Table 3
	LEL (organ-level)	RMSE: 0.41–0.68 log10-(mg/kg/day) mean RMSE across organ-level LELs^f^: 0.59 ± 0.09 log10-mg/kg/day	58–364, depending on target organ	151–1,353 studies	Paul Friedman et al. (2023) Fig 3/Supp File 3
Carcinogenicity (chronic testing) OECD TG 451^c^	TD50	*R* ^e^: 0.63^g^	121	70 studies	Gottmann et al. (2001)
Neurotoxicity	Motor activity Motor activity (across time) LOEL	Intralaboratory between subject control CV: 18.9%–30.7% Intralaboratory between subject control CV across time: 9.6%–26.2% LOEL-ratio range motor activity = 1–6^h^	1 (vehicle) 9	variable methods (cage configurations, rat strains, sex, age, test duration, interval-duration, housing conditions) in six laboratories	Crofton et al. (1991) Table 3
(DNT)	Motor activity (Negative control) Startle response (Negative control)	Intralaboratory CV: 20%–140% Intralaboratory CV: 20%–110%	NA	NA	Moser et al. (2016)
(DNT)	Brain morphometry Brain weight	Interlaboratory CV: 5%–30% Interlaboratory CV: 4%–12%	NA	12 studies, 7 labs 22 studies, 10 labs	Crofton (2001)

^a^ Replicate of same chemical.

^b^ Numbers indicate number of studies unless otherwise specified.

^c^ OECD, test guideline numbers represent the assay focus, however, several of the references include aggregated datasets including “guideline-like” studies with minor variations from the TG.

^d^ Upon exclusion of five most variable chemicals (SD, were reported per chemical; the highest SD, is reported as an upper bound).

^e^ Bootsrapping of all LD50 values 5,000 times yielded a representative MAD, the margin of uncertainty was then calculated as ±2.5 x the representative MAD.

^f^ Values based on multilinear regression modeling.

^g^ Correlation in the TD50 between data sources in the Carcinogenic Potency Database—the National Cancer Institute or National Toxicology Program *versus* the general literature.

^h^ Work presented at the Society of Toxicology Annual Meeting in 2003 analyzed animal control data from 21 studies from 10 laboratories and concluded that “Further consideration of how to reduce variability of these motor activity measurements is warranted” (Raffaele et al., 2004).

Definitions–CV, coefficient of variation; DNT, developmental neurotoxicity; LD50, dose resulting in lethality in half of test animals; LEL, lowest-effect level; LOEL, lowest observed effect level; LLNA, local lymph node assay; MAS, maximum average score; NA, not available; SD, standard deviation; TD50, dose resulting in tumors in half of test animals.

Quantifying replicability was noted as a significant challenge in most retrieved publications. Animal-based guideline studies have largely not been subjected to a validation process, and have instead been adopted based on historical precedent, providing few points of reference for comparison (Oyetade et al., 2023). Over the years there have been updates to some guidelines and more stringent GLP requirements have been introduced but insufficient data are available at this time to robustly evaluate the impact of these changes on variability; as such all analyses summarized herein aggregate all historical results into one compendium for analysis. Furthermore, it should be noted that testing data for all chemicals tested were summarized together as cheminformatics evaluation to dive into difference in chemical class variability per study have not been conducted herein (though some referenced studies have done cursory evaluations of chemical structure impact on variability for some endpoints (Karmaus et al., 2022; Pradeep et al., 2017; Ly Pham et al., 2020), often due to insufficient data for robust evaluation. Thus, replicability evaluations summarized herein relied upon replicate testing of single test articles (no formulations or mixtures). As noted above, we focused our analysis on two major types of replicability: replicability of an outcome, i.e., categorical (Table 3) and replicability of continuous data (Table 4).

### 3.1 Categorical replicability

Categorical replicability analyses focus on study types with a categorical interpretation, which can be either a binary outcome of positive or negative or assignment to a hazard classification. Study types with binary classification endpoints included in our review are endocrine assays (Hershberger and uterotrophic), genotoxicity studies, carcinogenicity studies, and DNT studies (Table 3). The Hershberger and uterotrophic assays gave similar degrees of replicability: 72% and 74%, respectively (Kleinstreuer et al., 2016; Browne et al., 2015). For carcinogenicity, the two prior variability evaluations yielded different outcomes: 65%–86% replicability when comparing between sexes of a single species, or 36%–74% replicability between different species (rat and mouse) (Huff et al., 1991; Haseman and Lockhart, 1993).

More complex categorical analyses were conducted for studies using multicategory hazard classification schema to assign chemicals categories based on quantitative or qualitative study results. As noted above, our evaluation of this aspect of replicability was limited to studies using the GHS (the most common classification scheme for chemical hazard categorization) and the EPA categorization scheme (specific to endpoints of interest for the EPA). These systems have different category cutoffs and varying numbers of categories.

Multicategorical replicability was evaluated using conditional probabilities where possible, gathered from retrospective variability analyses or calculated based on available study information. As a point of reference, assignment of categories by random chance would equate to 50% replicability for a binary categorization scheme or 33% and 25% for systems of three and four categories, respectively. Endpoints with such data included ocular irritation/corrosion, dermal sensitization, dermal irritation/corrosion, rat acute oral lethality, and rat acute inhalation lethality (Table 3). Replicability was generally higher for categorization systems with fewer categories. For genotoxicity studies, the replicability of three categories (positive, negative, or ambiguous outcomes) varied widely, ranging between 23% and 78%, depending on the TG and its protocol variants (Raitano et al., 2026). It was rare to observe replicability above 75% across many study and endpoint types, especially for complex or targeted endpoints such as organ-specific effects. Depending on how replicate studies were aggregated across study type and species, the concordance of any target organ effects for subchronic and chronic repeated dose studies ranged from 38.5% to 90% per organ and the frequency of positive findings (Hoffmann et al., 2005).

For dermal and ocular study types, increased replicability was observed for categories representing the lowest and highest toxicities, suggesting robust results when toxicity is either absent or overt. This is most notable in the Draize rabbit eye irritation test for which the GHS Not Categorized classification was replicated as much as 100%, while the GHS Category 2B classification, representing mild irritation, had a conditional probability of only 16% replicability (Luechtefeld et al., 2016; Barroso et al., 2017). Given the low replicability for such mid-categories (e.g., GHS categories representing hazard of mild to moderate ocular irritation), subsequent studies were conducted to evaluate the impact of combining these categories to determine whether the replication improved with grouping. When authors collapsed GHS Categories 2A and 2B for ocular irritation, replicability was improved to as high as 71% or 88% in two independently conducted analyses (Barroso et al., 2017; Adriaens et al., 2014). Two evaluations of dermal sensitization data conflicted with the increased replicability seen for fewer categories (Dumont et al., 2016; Hoffmann et al., 2005). One study that considered reproducibility of three GHS classifications found Not Classified outcomes to have 52% replicability, while another study that considered five classifications found these outcomes to have 80% replicability. The discrepancy may be explained based on the analysis approach used: the study finding 52% replicability was based on any chemical for which two or more studies were available, and all categories were assigned in a normalized approach with equal study weights. The study finding 80% replicability, in comparison, was found for chemicals with three or more studies and a single categorization determined based on the majority outcome. These findings underscore the importance of methods and review assumptions as well as curation protocols for retrospective analyses.

### 3.2 Quantitative replicability

The replicability of continuous numeric endpoints was reported using different quantitative approaches. Retrospective consideration of all of these disparate reported metrics is particularly important because most of these studies were not subject to replicability analysis for a range of chemicals prior to acceptance of the relevant TGs. Variability among quantitative endpoints was evaluated for ocular irritation, dermal sensitization, acute oral lethality, subchronic/chronic repeated dose toxicity, and carcinogenicity studies (Table 4). For acute lethality, variability analyses were available for both rat and mouse studies. Studies using either species showed similar degrees of variability when evaluated by the same authors (Hoffmann et al., 2010). Where possible, CVs were provided, but these were not available for all study outcomes due to a lack of reported data. Other quantitative metrics describing dispersion of the data included SD and a margin of uncertainty based on the median absolute deviation (MAD). Some variability analyses constructed models using study metadata to quantify the variance in replicate studies; these studies reported RMSE as a measure of spread of the predicted values from the true values, and *R* ^2^ as the amount of variance in the data explained by the model. When the model mean prediction is the same as the data mean, the RMSE is equal to the SD of the residuals. We have reported SD, margin of uncertainty, and RMSE in the same units as the data, whereas *R* ^2^ is expressed as a proportion of the variance explained by the model or variable (Table 4). Importantly, maximal *R* ^2^ attainable by using curated study metadata to explain variance is limited by unexplained variance due to undocumented parameters (i.e., experimental factors that were not collected with the study or not curated consistently) or inherent biological variance (Ly Pham et al., 2020; Kleinstreuer et al., 2016; Dumont et al., 2016; Paul Friedman et al., 2023; Lubet et al., 2018; Ashby, 2002; Crofton et al., 1991).

Although these different metrics measure different statistical observations of the data or models of the data, examining the set of statistical metrics revealed multiple high-level findings. Inter- and intralaboratory CV values were available for ocular irritation with comparable CV values ranging between 40% and 60% (Weil and Scala, 1971; Earl et al., 1997; Cormier et al., 1996; Blein et al., 1991). Single-dose acute studies demonstrated less dispersion of effect-level values, likely the product of study design (e.g., limit tests at 2000 mg/kg), with the margin of uncertainty equal to 0.25 log10-mg/kg in one evaluation (Karmaus et al., 2022) and the SD falling below 0.42 log10-mg/kg for most studies in another evaluation (Hoffmann et al., 2010). Repeat-dose studies demonstrated greater dispersion of replicate values that typically approached ±0.5 log10-mg/kg/day, depending on how the dispersion was quantified. The linear correlation between 50% tumorigenic doses between two separate sources of carcinogenicity studies showed an *R* ^2^ of 0.63 (Cormier et al., 1996), suggesting major differences in study conduct, curation, and/or biological observations. In the aggregate, estimates of variance suggested large amounts of spread in replicate study data for quantitative endpoints. Linear correlation of values related to carcinogenicity (Gottmann et al., 2001) and the amount of variance in replicate oral repeat-dose study toxicity values explained by multilinear regression modeling (Ly Pham et al., 2020; Paul Friedman et al., 2023) suggest some upper bound exists in the amount of variability in replicate toxicity values that can be explained by study metadata, which likely approaches 60%–70%.

One evaluation of replicability for behavioral endpoints assessed as part of neurotoxicity studies indicated good replicability for motor activity studies, with a lowest-observed-effect level maximum-to-minimum ratio range of one to six for nine positive control compounds tested within six laboratories (Crofton et al., 1991). Replicability of DNT motor activity was reported for negative controls showing CVs ranging from 20% to 140% (Moser et al., 2016). Despite the broad variability, these are notable data as negative controls are rarely reported. No other studies were available that could provide replicability estimates for this or other behavioral methods. Several abstracts presented at Society of Toxicology Annual Meetings between 2001 and 2005 (which were not peer-reviewed) indicated incomplete reporting and incomplete positive control data in evaluations of startle response data (Sette et al., 2004) and learning and memory tests (Raffaele et al., 2004), such that the considerable within- and between-lab variability could not be adequately analyzed for these endpoints. One of these, a 2004 evaluation of motor activity measurements, indicated that, “*Further consideration of how to reduce variability … is warranted”* (Raffaele et al., 2004). A qualitative retrospective analysis of DNT studies reported that few laboratories (3/16) provided usable positive control data (Crofton et al., 2004), with studies lacking such data thus being uninformative for replicability estimates. However, negative control CV values for parameters of motor activity or startle response may provide some indication of the replicability of study results. Typically, CV values for such readouts range between 20% and more than 100%, depending on the laboratory, test conditions, and animal age (Moser et al., 2016). These high and variable CV values indicate that significant differences between control and dose groups may be recognized in some labs and test conditions but not in others. In contrast, as reported in a 2001 Society of Toxicology Annual Meeting presentation, CVs among brain weights and brain morphometrics were reported to be significantly lower, ranging from 4% to 12% and 5%–30%, respectively (Crofton, 2001).

## 4 Discussion

With the recent spotlight on variability in the biomedical sciences (National Academies of Sciences E, 2019; Baker, 2016; Begley and Ioannidis, 2015; Poland et al., 2014), and the shift to NAMs for which comprehensive performance metrics are being generated as part of method development and validation (van der Zalm et al., 2022; Gamble et al., 2025; Foley et al., 2024; Miedel et al., 2025), it is more important than ever to compile variability metrics for available *in vivo* data. Much has been written about the challenges of scientific experimental reproducibility and variability, with multiple analyses specifically quantifying bioassay replicability. A Nature survey of nearly 1,600 scientists across multiple fields found that more than 70% of respondents had tried and failed to reproduce a previously published study from another scientist (Baker, 2016). Recent assessments of studies in psychology have suggested a 40% rate of replicability for these studies, with other evaluations suggesting a dismal 10% rate of replicability for cancer biology studies (Baker, 2016). This proposed “reproducibility crisis in science” (or “replicability crisis” based on definition of terms used herein) has garnered the attention of the general public and can impact the scrutiny applied to evaluating new approaches (Haven and Ioannidis, 2025).

Our review suggests replicability of *in vivo* toxicological guideline studies varies widely by study type, endpoint complexity, and classification approach depending on whether study designs employ binary or multicategorical outcomes or continuous endpoints. While optimizing assays to achieve low variability is fundamental to scientific investigation, it should be noted that variability should not be equated with lack of validity. Biology is inherently variable, and therefore variability in *in vivo* assays is not necessarily indicative of a poorly performing test method. However, it is imperative to properly integrate this information as uncertainty into any interpretation of the study data and associated analysis pipelines. Retrospective quantification of assay variability can establish acceptable levels for varying contexts of use and help identify which sources of variability lack adequate controls to ensure that the most robust available science is applied for toxicological safety assessments. The variability assessments summarized herein highlight a particular point of emphasis associated with test method evaluations: since *in vivo* tests used as a benchmark are not fully replicable, we cannot expect NAMs to have greater precision than a replicate *in vivo* study would. Thus, characterizing traditional guideline toxicological study variability can help establish baseline expectations for the use of NAMs.

### 4.1 Distinguishing sources of variability

It is important to distinguish between quantified variability, sources of variability, and uncertainty about variability. For evaluating traditional *in vivo* toxicological assays, we must consider the effects of study conduct on both derivation of quantitative points-of-departure (hazard characterization) and interpretation frameworks (hazard identification, e.g., classification) for replicability. It is also important to note that different group size and statistical power between studies may explain limited replicability of study outcomes. Previous investigations have revealed that variability can stem from numerous sources related to either the assay protocol or study design variations, including elements such as animal strain, diet, and vehicle used (Lubet et al., 2018; Ashby, 2002). For example, a review focusing on variability in genotoxicity studies conducted a multivariate analysis to identify drivers of variability for both OECD TGs 474 and 475 across 31 chemicals with replicate data identifying strain and species as having the greatest contribution to variability (Raitano et al., 2026). Administration *via* injection as compared to the oral route has also been shown to increase the likelihood of a positive response in uterotrophic assays (Kleinstreuer et al., 2016), and lower variability is observed among studies that use the same vehicle (Dumont et al., 2016). Study parameters that can be customized such as species, dose spacing, and substance purity were found to contribute to more than half the total variance in organ-level lowest-effect levels (LELs) (Paul Friedman et al., 2023). This is particularly well-characterized for underlying physiological and metabolic differences across species, strain, and sex. For example, tumor incidence in the rat reproductive tract (You et al., 2002; Buelke-Sam et al., 1998) or the presence of thyroid tumors in male rats vs. female rats (Coperchini et al., 2025). In addition, one report indicated that endpoint selection in the Draize rabbit eye irritation test had a significant impact on the degree of variability: higher variability was seen when a GHS Category 2 classification was made based on conjunctiva effects without corneal involvement as compared to classification based on other drivers such as iritis (Adriaens et al., 2014). Protocol timepoints, such as the age of the animal, the timepoint following exposure, or timing of the response measurement, can also significantly impact study outcome (Lubet et al., 2018; Ashby, 2002). We must acknowledge that TGs are not strict standard operating procedures, recognizing that some flexibility in the study design is acceptable and within the scope of “guideline-like” study conduct. However, even where the source of the variability may be explained, the data variability may remain an uncertainty since it is difficult to understand which protocol variant is most relevant for the human population.

Addressing inherent biological variability is even more complex, but can be considered to be derived from four general factors: physiological, genetic, ontogenetic, and exposomic (Kreutz et al., 2024). These sources of variability have been quantified to some extent but require further characterization. For example, with regard to chemical toxicokinetics, studies have found that variability estimates usually, but not always, fall within the acceptable 10X uncertainty factor for some chemicals and lifestages, with many uncertainties remaining (Quignot et al., 2021; Dorne et al., 2001; Kasteel et al., 2020; Darney et al., 2020; Di Consiglio et al., 2021; Wetmore et al., 2015; Strikwold et al., 2017). There are also uncertainties regarding the generalizability of animal studies to human and ecosystem outcomes due to differences such as species, exposures, and endpoints. Both animal models and *in vitro* NAMs present challenges for generalizability of model outputs to humans, with neither paradigm fully representing the toxicokinetic and toxicodynamic processes present in humans. Rodent-based studies must be extrapolated to human equivalent doses for human health risk assessment using a set of assumptions regarding dosimetry, toxicokinetics, and applicability of targets in rodents for targets in humans. Human-based NAMs are typically isolated cell-based or protein-based assays that require extrapolation to the human body, including toxicokinetic and metabolic considerations, such as tissue-specific metabolism.

### 4.2 Considerations for addressing data gaps and future work

Notable data gaps in our retrospective analysis include reproductive and developmental toxicity study types and toxicokinetic studies as well as variability among controls for these studies, which are rarely reported. We also note that the different analyses summarized did not all represent unique data sets, as some of the larger replicability evaluations mined the same source databases, resulting in overlapping representation of primary data. However, as our goal was not to directly compare each variability study, we felt that some redundancy was acceptable as we sought to more comprehensively characterize variability. More specifically, even variability estimates reported from the same datasets may differ due to the filtering steps and statistical approaches applied. One such case is for the vehicle used: while some analyses provided separate estimates of variability for the same vehicle (Adriaens et al., 2014), most did not consider vehicle as a factor in their analyses.

Results summarized here are likely not unique for mammalian toxicology and are expected to be consistent across ecotoxicology studies as well. For example, 50% lethality or moribundity concentrations (EC50s) in acute fish toxicity studies performed according to OECD TG 203 may vary over several orders of magnitude, with variation only partly accounted for by the use of the 11 different fish species allowed in the TG. Similar levels of variability are observed in EC50 data from acute daphnid studies conducted according to TG 202 (Schür et al., 2025). Extending this review to other such endpoints could prove useful to better characterize current toxicological test methods. However, it is necessary to acknowledge that performing literature searches and compiling comprehensive, harmonized, and robust datasets required for retrospective analyses is a laborious task that will never capture all existing data and important metadata.

To more readily access individual or collated study data for conducting retrospective evaluations, databases of *in vivo* results have been developed and are routinely being updated. Some of these from the United States include the EPA’s ToxRefDB (Feshuk et al., 2023a) and ToxValDB (Wall et al., 2025), the National Toxicology Program’s Chemical Effects in Biological Systems database (CEBS), and the National Institutes of Health’s Integrated Chemical Environment (https://ice.ntp.niehs.nih.gov/). Additional resources from Europe include the European Chemical Agency’s (ECHA) IUCLID database (https://iuclid6.echa.europa.eu/) and the European Food Safety Authority’s (EFSA) Open FoodTox (https://www.efsa.europa.eu/en/microstrategy/openfoodtox). While each of these databases include summary endpoint metrics per chemical (e.g., LEL, NOAEL, LD50, *etc.*), a few of the resources (namely, ToxRefDB and CEBS) also have prioritized supporting detailed concentration-response data (e.g., response values per testing concentration) which can further support more granular retrospective evaluations. Of course, each of these databases has varying numbers of chemicals and studies, and compilation of a dataset for robust retrospective evaluation requires careful characterization and curation. Future retrospectives using data from such databases should be performed to gain further insights into reproducibility of study types or routes of administration that we were unable to consider here, e.g., reproductive and developmental, inhalation, or dermal repeat-dose toxicity studies. Investigation of whether reproducibility has improved over time, e.g., due to more stringent GLP requirements and given TG revisions, could also be considered. The increasing requirements for findability, accessibility, interoperability, and reusability of data (so-called “FAIR” principles) should help to facilitate future evaluations of study variability.

### 4.3 Implications for risk assessment and NAMs

Both traditional animal models and human-based NAMs can be used to derive protective points-of-departure for human health risk assessment. In recent work examining pharmaceuticals, the difference between preclinical animal and NAM-based points-of-departure in predicting doses at which human toxicity was observed were compared (Weitekamp et al., 2025). NAM-based values were consistently lower than rodent-based values. For values from rodent studies converted to a human equivalent dose to be protective of human adverse effect levels for at least 95% of pharmaceuticals in the dataset they needed to be divided by a composite factor of at least 100. However, for human NAM-based values converted to human equivalent doses to provide a similar level of protectivity, they needed to be divided by a factor of only 10. Further work is required to integrate uncertainties and variability to account for biological factors that are critical for understanding how NAM-based values can inform toxicity values when compared to traditional methods.

It is possible that study variability is accounted for in the conservatism of uncertainty factors applied when deriving toxicity values, such as for extrapolation from subchronic to chronic values or database uncertainty. That said, it should be emphasized that study variability, and the specific uncertainty introduced by study variability, is not typically directly recognized. In recent work to rapidly derive database-calibrated oral toxicity values, study variability is included in quantification of uncertainty in deriving these toxicity values (Aurisano et al., 2023; Harrill et al., 2026). In many regulatory paradigms, regulators must develop a weight-of-evidence strategy for decision-making, and this can include review of databases with heterogenous data and grouping or read-across approaches for the reuse of data from similar compounds. Therefore, the gross estimates for *in vivo* data variability provided in this review may be useful when considering current regulatory practice. Next-generation risk assessments focus on establishing scientific confidence through a weight-of-evidence approach that considers reproducibility and technical characterization in addition to biological relevance, mechanistic understanding, and fitness for purpose (van der Zalm et al., 2022; ICCVAM, 2024). Such considerations support innovation using modern science and facilitate regulatory acceptance of NAMs in safety assessments that ensure the protection of human health and the environment. However, building confidence in new test systems requires demonstration of robust, consistent, and interpretable outcomes. One suggested first step, which is already frequently being performed, would be to confirm assay performance in terms of replicability and minimal variability, relative to animal TG data variability. For example, variability can be more tightly controlled through rigorous and well-defined assay development frameworks for NAMs (ICCVAM, 2024). In many cases, studies involving NAMs include much higher replicate counts and internal positive controls. In addition, reporting of variability measures is becoming a common practice. Additional databases of *in vitro* data including PubChem (Kim et al., 2025) and the EPA’s invitrodb (Feshuk et al., 2023b) contain some performance metrics and individual replicate data that can be leveraged for further retrospective evaluations of NAM variability. Such analyses of both biological and technical replicability can of course be included among the metrics for consideration of a NAM as “better than” traditional *in vivo* approaches. Building additional validation approaches beyond those that rely solely on direct comparisons to legacy animal tests and qualifying expectations of human-based NAMs to recapitulate rodent findings reflects a growing recognition that fundamentally different principles should be considered when evaluating NAMs.

## 5 Conclusion

Here we provide a summary of variability analyses for *in vivo* TGs applied for human safety assessments. While such variability data are necessarily uncertain, they are nevertheless useful as legacy reference data and considering the current regulatory practice for data acceptance and weight-of-evidence assessment. We argue that benchmarking of NAMs must include integration of *in vivo* bioassay replicability. This means that the validation of NAMs for human toxicology should rely on multiple factors, including estimates of variability of NAM data; biological and mechanistic relevance of the NAM assay for the human target or process; an assessment of how the NAM data may achieve similar or better protection of human health when compared to animal study data; and the specific regulatory purpose of the data (van der Zalm et al., 2022). We hope the summarized variability metrics herein will help inform the regulatory acceptance of NAMs, particularly in regard to facilitating comparisons of replicability for NAMs vs. for historically used *in vivo* TGs. This is an important comparison to enable as NAMs may need to demonstrate “equivalent or better” assay performance.

## Data Availability

Publicly available datasets were analyzed in this study. This data can be found here: References to the data is included in the manuscript.
